# P-1855. Enhancing Patient Outcomes in Outpatient Parenteral Antimicrobial Therapy (OPAT): The Role of the Multidisciplinary Team

**DOI:** 10.1093/ofid/ofaf695.2024

**Published:** 2026-01-11

**Authors:** Eka Beriashvili, Maribel Galeano, Ryan Rosen, Paula A Eckardt, Sheila Montalvo, Alecia Muwonge, Dana J Holger, Garrett Van Ostran

**Affiliations:** Memorial Healthcare System, FortLauderdale, FL; Memorial Healthcare System, FortLauderdale, FL; Memorial Healthcare System, FortLauderdale, FL; Memorial Healthcare System, FortLauderdale, FL; Memorial Hospital System, Cooper City, FL; Memorial Healthcare System, FortLauderdale, FL; Nova Southeastern University, Fort Lauderdale, Florida; Memorial Healthcare Systems, Hollywood, Florida

## Abstract

**Background:**

Outpatient Parenteral Antimicrobial Therapy (OPAT) facilitates IV antibiotic administration in the outpatient setting, enhancing patient satisfaction and reducing healthcare costs. Continuous monitoring and comparison to national benchmarks support safe, effective care and drive quality improvement. Memorial Physician Group–Infectious Disease (MPG-ID) and Memorial Home Infusion (MHI) launched a formal OPAT service at Memorial Healthcare System (MHS) in February 2022, which has since expanded to meet growing demand.

Objective: To describe the demographics, clinical characteristics, and outcomes of patients referred for OPAT at a community hospital.

**Figure:**
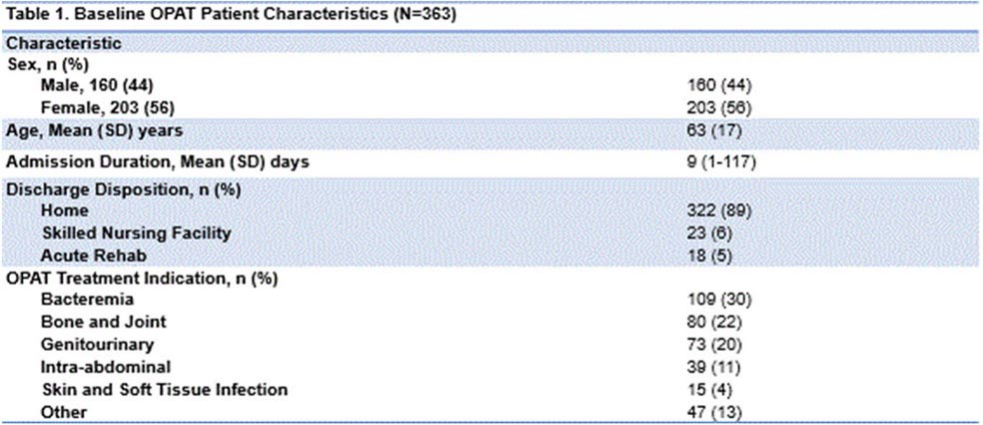

**Figure:**
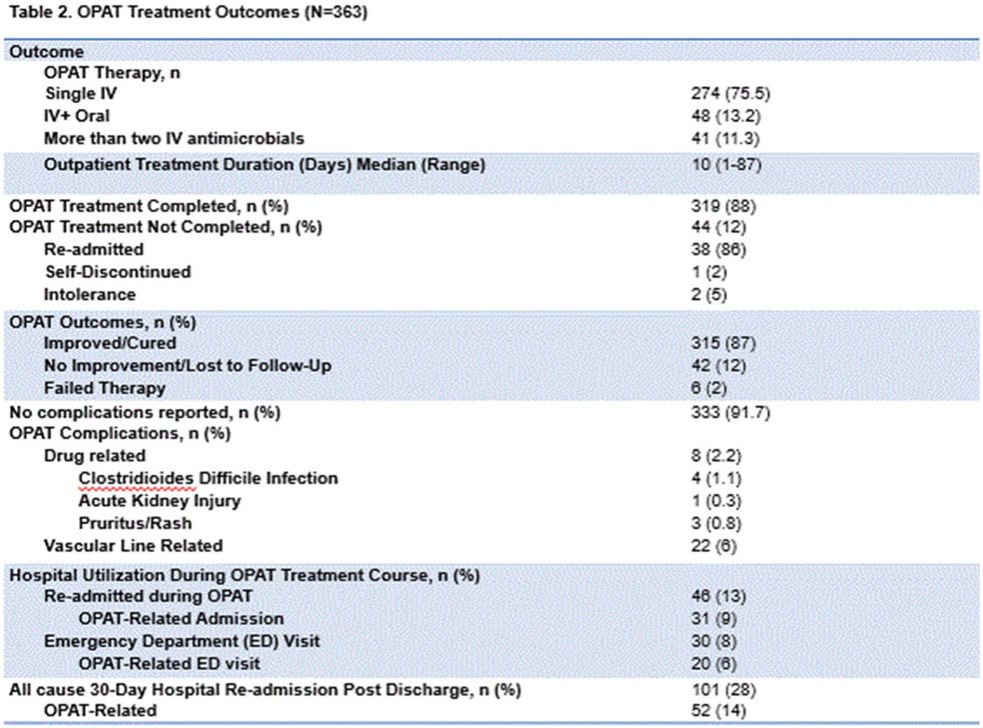

**Methods:**

This single-site retrospective chart review included MHS patients referred for OPAT at the MPG-ID clinic between Aug 1, 2024, and Jan 31, 2025. Patients were evaluated by the MPG-ID team and followed until OPAT completion or discontinuation. An OPAT pharmacist and nurse managed labs and therapy five days a week. Key metrics included 30-day healthcare utilization and treatment outcomes. Patients were excluded if they received full inpatient IV therapy, left against medical advice, were discharged to hospice, on chronic suppressive therapy, or transferred to other ID services.

**Figure:**
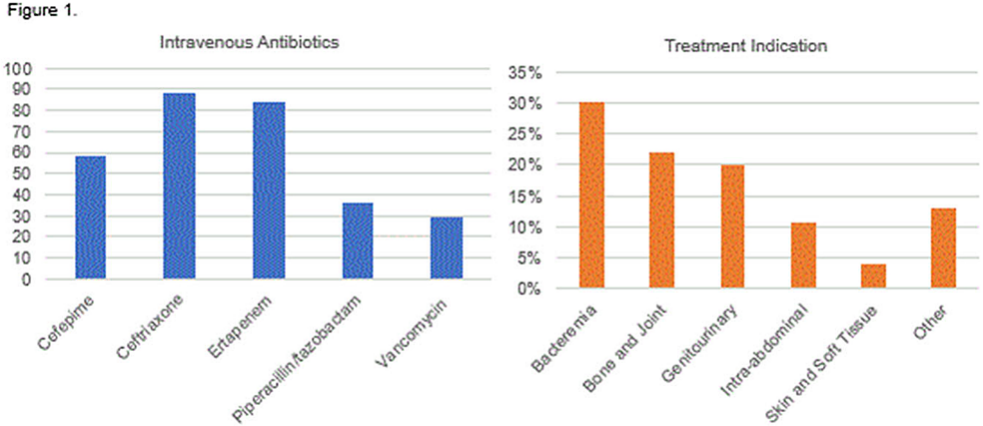

**Results:**

Of 414 OPAT referrals, 363 met criteria for analysis. 89% of OPAT orders were reviewed within 72 hours (Table 1). Clinic follow-up occurred in 58% of patients; 88% completed treatment, with an 87% infection resolution rate. The 30-day OPAT-related readmission rate was 14%. Most received a single IV regimen (75.5%). Treatment failure and ADR rates were 2% and 2.2%, respectively - both lower than national averages (Table 2). Bacteremia (30%), bone/joint infections (22%), and genitourinary infections (20%) were the most common indications (Figure 1). A total of 5,369 outpatient treatment days were recorded, with estimated cost savings of $10.7M–$16.1M.

**Conclusion:**

Our OPAT program demonstrates strong clinical outcomes, low adverse event rates, and alignment with national benchmarks. Early pharmacist review and ongoing multidisciplinary care contributed to enhanced safety, antimicrobial stewardship, and cost efficiency.

**Disclosures:**

All Authors: No reported disclosures

